# An EMT-based risk score thoroughly predicts the clinical prognosis, tumor immune microenvironment and molecular subtypes of bladder cancer

**DOI:** 10.3389/fimmu.2022.1000321

**Published:** 2022-09-23

**Authors:** Zicheng Xiao, Zhiyong Cai, Dingshan Deng, Shiyu Tong, Xiongbing Zu

**Affiliations:** ^1^ Department of Urology, Xiangya Hospital, Central South University, Changsha, China; ^2^ National Clinical Research Center for Geriatric Disorders, Xiangya Hospital, Central South University, Changsha, China

**Keywords:** bladder cancer, prognosis, tumor microenvironment, molecular subtype, immunotherapy

## Abstract

**Background:**

Epithelial mesenchymal transition (EMT) is closely related to the occurrence, development, metastasis and antitumor immunity of tumors. However, comprehensive studies correlating EMT and prognosis, tumor microenvironment (TME) and molecular subtypes of bladder cancer (BLCA) are lacking.

**Methods:**

TCGA-BLCA was chosen as our training cohort, while Xiangya cohort, GSE13507, GSE48075 were selected as our validation cohorts. Prognostic genes were screened out using univariate Cox analysis and the least absolute shrinkage and selection operator (LASSO) algorithm. Then we developed an EMT risk score based on these prognostic genes and systematically correlated the risk score with prognosis, TME and molecular subtypes of BLCA.

**Results:**

Based on EMT related genes, we developed two different EMT patterns, named EMT cluster 1 and cluster 2, and found that cluster 2 showed a worse prognosis and an inflammatory TME phenotype. For personalized prognosis and TME phenotypes predicting, we developed and validated an EMT-based risk score by 7 candidate genes (ANXA10, CNTN1, FAM180A, FN1, IGFL2, KANK4 and TOX3). Patients with high EMT risk scores had lower overall survival (OS) with high predictive accuracy both in the training cohort and validation cohort. In addition, we comprehensively correlated the EMT risk score with TME and molecular subtype, and found that high EMT risk score suggested higher levels of immune cell infiltration and more inclined to present the basal molecular subtype. It was noteworthy that the same results also appeared in the validation of Xiangya cohort.

**Conclusions:**

EMT related genes play an important role in tumor progression and immunity in BLCA. Our EMT risk score could accurately predict prognosis and immunophenotype of a single patient, which could guide more effective precision medical strategies.

## Introduction

Bladder cancer (BLCA) was the second most common urothelial malignancy and the 10th most common cancer worldwide, with nearly 550 thousand new diagnoses and 200 thousand new deaths each year (1, 2). According to whether the tumor invaded the detrusor muscle, BLCA could be divided into muscle invasive bladder cancer (MIBC; T2, T3, and T4) and non-muscle invasive bladder cancer (NMIBC; Tis, Ta, and T1) (3). About a quarter of newly diagnosed BLCA patients are MIBC, and about 15% to 20% of NMIBC patients will progress to MIBC. MIBC is characterized by high progression and metastasis, and even after radical surgery, its 5-year survival rate is still very poor (3). Immunotherapy played an important role in patients with advanced and intolerable cisplatin chemotherapy (4). Nevertheless, many patients were not sensitive to immunotherapy (5), so it is of great importance to explore biomarkers that can predict the efficacy of immunotherapy.

Tumor microenvironment (TME) refers to the local homeostasis related to the occurrence, development and metastasis of tumor, which is composed of tumor cells and non-tumor cells in the process of tumor growth (6, 7). Immune cells are an important part of non-tumor cells, which participate in the composition of tumor immune microenvironment (TIME), and the interaction between these cells affects the progress and treatment response of cancer (8). Many previous studies had found that the difference of TIME was related to the immunotherapeutic effect of MIBC (9, 10). In addition, TME can be divided into inflammatory and non-inflammatory, while inflammatory TME was sensitive to immunotherapy and non-inflammatory tumor was not (11). Tumor infiltrating immune cells were the basis of antitumor effect, especially T cells. Studies had proved that infiltrating CD8 positive T cells was a favorable factor for immunotherapy response (12). Therefore, understanding the characteristics of TME and regulating the transformation of TME typing was a method with great potential to improve the immunotherapy response rate of MIBC.

Epithelial mesenchymal transition (EMT) is involved in a large number of cancer-related events, including cancer invasion, metastasis, cell death resistance and antitumor immunity. Recently, studies have shown that EMT is a gradual process through different cell states, and the changes of different EMT transition states are mediated by gene regulatory networks (GRNs), which control the specific gene expression program of each state (13). Studies had shown that EMT was closely related to immunity, such as affecting the sensitivity of tumor cells to macrophages and natural killer cells (14). Therefore, studying the interaction between EMT related genes and developing an integrated prediction model can provide more practical help for clinical cancer treatment. However, comprehensive studies about the expression signatures, immune microenvironment status, and predictive prognostic value of EMT related genes in BLCA had not been well defined. In this study, we aimed to comprehensively investigate the predictive value of EMT related genes in the prognosis, TIME and molecular subtypes of BLCA.

## Materials and methods

### Acquisition and preprocessing of BLCA datasets

#### Training Set

We selected 408 BLCA patients from the Cancer Genome Atlas (TCGA) database and downloaded their mRNA expression matrix and clinical information from Genomic Data Commons (GDC, https://portal.gdc.cancer.gov/) (15). The expression matrix included the fragments per kilobase of exon model per million mapped fragments (FPKM) and the count value, then we converted the FPKM into the transcripts per kilobase of exon model per million mapped reads (TPM) and integrated them with clinical information. After removing 5 patients with duplicate or incomplete follow-up information, 403 TCGA-BLCA patients were included in the training cohort.

#### Validation cohorts

Based on our previous research (16), we uploaded an RNA-sequencing (RNA-seq) cohort (GSE188715) based on 56 BLCA patients in Xiangya Hospital with complete survival information on the Gene Expression Omnibus (GEO) database, named Xiangya cohort. Similarly, we downloaded two external data sets from the GEO database (https://www.ncbi.nlm.nih.gov/geo/), then eliminated the cases with duplicate or incomplete survival information, and finally got another two validation cohorts, including GSE13507 with 165 samples and GSE48075 with 142 samples. The clinical information of these four datasets was summarized in **Supplementary Table S1**.

### Consensus clustering

We downloaded 200 EMT-related genes from the Molecular Signatures Database (MSigDB) (http://www.gseamsigdb.org/gsea/msigdb/cards/HALLMARK_EPITHELIAL_MESENCHYMAL_TRANSITION.html) (**Supplementary Table S2**). We used the consensus clustering function (parameter setting: maxK=5, Reps=1000, pItem=0.8, pFeature=1, distance=“manhattan”, clusterAlg=“pam”) in the “ConsuClusterPlus” R package to analyze the expression characteristics of these 200 EMT-related genes in TCGA-BLCA, and then developed different EMT expression patterns (17).

### Differentially expressed genes screening and functional analysis

We applied empirical Bayesian algorithm implemented in the “limma” R package to identify DEGs in different EMT expression patterns. We regarded the genes with absolute log2 fold change (| log2FC|) greater than 2.5 and adjusted p value less than 0.01 were as DEGs. Then, we conducted Gene Ontology (GO) and Kyoto Encyclopedia of Genes and Genomes (KEGG) analyses using the “ClusterProfiler” R package.

### Describing the tumor immune microenvironment of BLCA

We downloaded the activation levels of the 7-step Cancer Immunity Cycle from the tracking tumor immunophenotype (TIP) (http://biocc.hrbmu.edu.cn/TIP/) (18). Then, we used 5 different algorithms, including the single-sample gene set enrichment analysis (ssGSEA), MCP, quantiseq, TIMER and xcell, to estimate the infiltration level of several tumor infiltrating immune cells in TIME, and the gene set for individual immune cells calculating was collected in **Supplementary Table S3** (19). Moreover, we further described the TIME through the expression of 22 immune checkpoint inhibitor (ICI) genes, 18 T cell-associated inflammatory signature (TIS) genes and other immune cell effector genes, such as CD8^+^ T cells, dendritic cells (DCs), macrophages, natural killer (NK) cells and type 1 T helper (Th1) cells (**Supplementary Table S4**), as we summarized in our previous studies (20).

### The construction, verification and clinical application of EMT risk score

Based on the above EMT-related DEGs, we screened the genes with prognostic value by using univariate Cox analysis and the “survival” R package. Subsequently, the “glmnet” R package was used to implement the LASSO regression and further refine the prognostic EMT-related DEGs to develop the risk score. Moreover, based on the expression features of the final 7 genes that we extracted above, we constructed an EMT-related risk score using the “rfsrc” algorithm in the “randomForestSRC” R package, named EMT risk score.

Every patient got his own EMT risk score, and those who were higher than the median EMT risk score were divided into high risk score group, the others were low scores. We drew the survival curve and receiver operating characteristic (ROC) curve, and calculate the area under the ROC curve (AUC) to verify the predictive ability and accuracy of EMT risk score. The above verification methods were realized by The Kaplan-Meier (K-M) method and log-rank test implemented in the “survminer” R package and the timeROC function implemented in the “tROC” R package. In addition, we constructed a nomogram using clinical information related to prognosis and EMT risk score, and verified its predictive efficacy with the calibration curve.

### Prediction of molecular subtypes of bladder cancer by EMT risk score

Previous studies of our team had summarized the existing 7 molecular typing criteria for bladder cancer, including the TCGA, UNC, and Consensus systems, at the same time, we had realized the unification of the 7 classification methods based on two R packages of the “BLCAsubtyping” and “ConsensusMIBC” (20). In addition, the bladder cancer related pathways come from the study of Kamoun, A. et al. (21). (**Supplementary Table S5**). In order to guide the clinic more concisely and efficiently, we reclassified different molecular subtypes into “luminal” and “basal” subtypes.

### Statistical analysis

We used Pearson or Spearman coefficients to express the correlation between variables. We used T-test or Mann-Whitney U test to represent the difference between binary groups in continuous variables. We drew the survival curve and calculated statistical significance to reflect the prognostic outcome by using the K-M method and log-rank test. We identified the DEGs between different EMT expression patterns by using the empirical Bayesian method. The univariate Cox analysis and LASSO algorithm were used to narrow and refine the range of candidate genes for constructing EMT risk score. The univariate and multivariate Cox regression models were used to calculate the hazard ratio (HR) and independent prognostic values of EMT risk score. The EMT risk score was constructed by the random survival forest (RSF) model, and its accuracy was reflected by drawing the time-dependent ROC curve and calculating the AUC. The standard of statistically significant difference was set as P < 0.05, and the adjusted P values in differential expression analysis were obtained by FDR (false discovery rate) method. All the statistical tests were used two-side test. All the statistical analyses were performed by R software.

## Results

### Internal relevance and development of expression patterns of EMT-related genes

**Figure 1:**
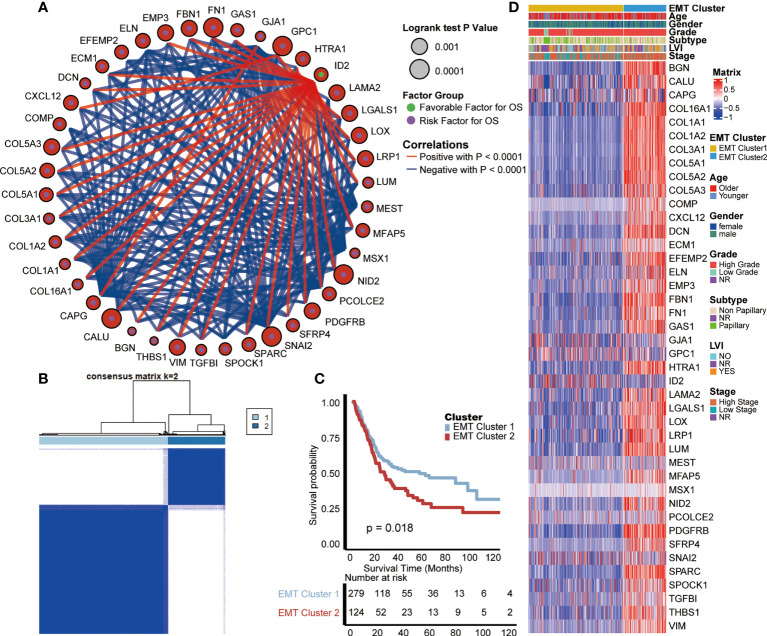
Internal relevance and development of expression patterns of EMT-related genes. **(A)** The interaction between EMT-related genes in BLCA. The size of the circle represented the p-value of overall survival (OS) calculated using log-rank test; The green and purple dots in the circle represented the favorable and risk factors for OS separately; Red and blue lines represented positive and negative correlations between EMT-related genes, separately. **(B)** The unsupervised cluster analysis based on all the 200 EMT-related genes; Light blue and dark blue lines represented EMT cluster 1 and 2, separately. **(C)** Kaplan-Meier plot of OS between two EMT-based patterns; Light blue and red lines represented EMT cluster 1 and 2, separately. **(D)** The differences of expression of prognosis related EMT genes and clinical information between two EMT-based patterns; Yellow and blue lines represented TNF cluster 1 and 2, separately.

We selected 42 EMT-related genes (P < 0.01) which were significantly related to the prognosis of BLCA from the HALLMARK_EPITHELIAL_MESENCHYMAL_TRANSITION gene set by univariate analysis, and comprehensively showed their association, interaction and prognosis (**Figure 1A**). There is a close relationship between EMT-related genes. Interestingly, most of those genes except ID2 have negative correlations with each other, and all suggest a poor prognosis of BLCA. Therefore, based on all the 200 EMT-related genes, we used the “ConsenseClusterPlus” R package for unsupervised cluster analysis, and found that it was most appropriate to divide TCGA-BLCA patients into two modes (**Figure 1B**).

These two patterns were named cluster 1 and cluster 2, and survival analysis showed that cluster 1 had a better prognosis than cluster 2 (p = 0.018, **Figure 1C**). Moreover, we analyzed the differences of expression of prognosis related genes and clinical information between these two EMT-related patterns. As shown in the heat map (**Figure 1D**), most of the genes strongly suggesting poor prognosis in the EMT pathway are highly expressed in cluster 2. As for clinical information, compared with cluster 1, patients in cluster 2 were characterized by older age, higher grade and stage, and higher proportion of positive lymphatic vessel invasion (LVI).

### Functional analysis of EMT-related patterns and association with TIME

**Figure 2:**
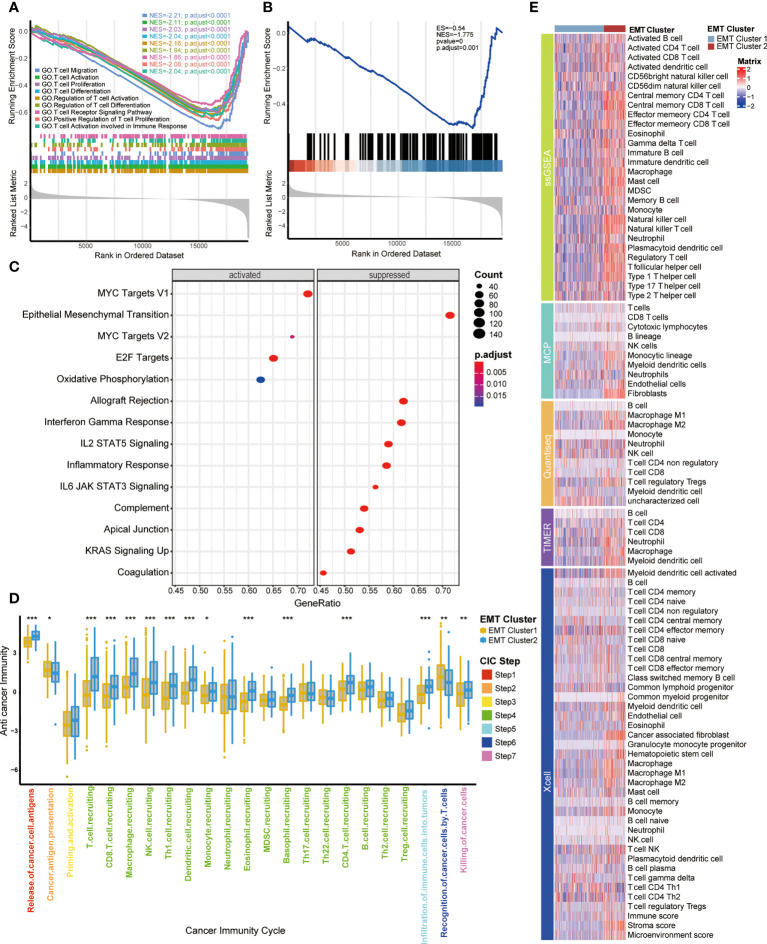
Functional analysis of EMT-related patterns and association with TIME **(A, B)** GSEA enrichment analysis of the status of KEGG and GO pathways in two EMT Cluster groups; EMT Cluster1 vs EMT Cluster2. **(C)** The difference on the hallmark gene sets between two EMT Cluster groups; EMT Cluster1 vs EMT Cluster2. **(D)** The different levels of anti-cancer immunity between two EMT-based patterns; Yellow and Blue lines represented EMT cluster 1 and 2, separately. *P < 0.05; **P < 0.01; ***P < 0.001; ns, not statistically significant. **(E)** Describe the difference of immune cell infiltration in TME between two EMT related patterns using five different algorithms, including ssGSEA, MCP, Quantiseq, TIMER and Xcell; Dark purple and light purple lines represented TNF cluster 1 and 2, separately.

Previously, we observed the survival difference between cluster 1 and cluster 2, so we further explored the biological significance behind them through the signal pathway, and then were surprised to find that the differential pathway between them was significantly enriched in T cell related pathways. As shown in GO and KEGG analysis (**Figures 2A, B**, **Supplementary Table S6**), compared with cluster 2, cluster 1 had significantly lower enrichment scores (ES) in T cell related pathways, including T cell migration, positive regulation of T cell proliferation, T cell activation and so on, which indicates that cluster 2 had a higher level of T cell enrichment than cluster 1. In addition, the ESs of Hallmark pathways in two clusters were prominently different (**Figure 2C**, **Supplementary Table S6**), indicating that the two EMT expression patterns had different biological functions. Cluster 1 was characterized by MYC targets V1 and V2, and E2F targets pathways enrichment, meanwhile, cluster 2 was characterized by allograft rejection, interferon gamma response and EMT pathways enrichment. By analyzing the pathways, we found that there were immune differences between the two EMT clusters, so we further compared the infiltration levels of immune cells. Chen et al. proposed a seven-step immune process, named Cancer Immunity Cycle (22); the anti-tumor immune response must start, develop and expand these series of gradual events in order to effectively kill cancer cells. Apparently, the activation in these main steps of Cancer Immunity Cycle, such as step 1 (release of cancer cell antigens), step 4 (T cell recruiting, CD8 T cell recruiting, macrophage recruiting, NK cell recruiting, Th1 cell recruiting, dendritic cell recruiting, CD4 T cell recruiting), step 5 (infiltration of immune cells into tumors), step 7 (killing of cancer cells), were significantly higher in cluster 2 than cluster 1 (**Figure 2D**). This result indicated that BLCA patients with cluster 2 had an inflamed TIME.

Moreover, we used five different algorithms to explore the regulatory role of EMT related patterns in TME (**Figure 2E**), including ssGSEA, MCP, Quantiseq, TIMER and Xcell. Cluster 2 was characterized by higher immune cell infiltration, including activated B cells, memory B cells, activated CD4 T cells, activated CD8 T cells, memory T cells, NK cells, macrophages, Th1 cells and higher immune score and stroma score. That further confirmed that cluster 2 may be an inflammatory phenotype with active TIME and more sensitive to immunotherapy.

### Construction of EMT risk score and survival verification of internal and external cohorts

Based on the expression characteristics of EMT-related genes, BLCA patients can be divided into two clusters with opposite prognosis and TIME. Therefore, further developing a quantitative score based on EMT related genes is of great significance for the individualized evaluation of BLCA.

**Figure 3:**
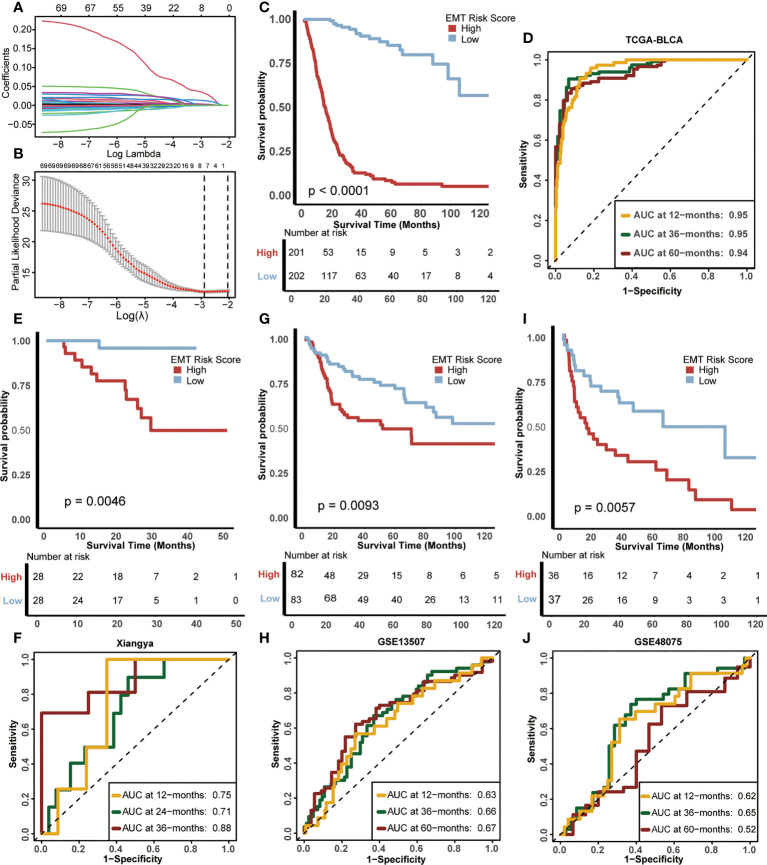
Construction of EMT risk score and survival verification of internal and external cohorts. **(A)** Coefficients of EMT-related differential expressed genes (DEGs) with prognostic value are shown by lambda parameter. **(B)** Partial likelihood deviance versus log (lambda) drawn by LASSO algorithm and 10-fold cross-validation. **(C)** Kaplan-Meier (K-M) plot of OS between EMT risk score groups; Red and light blue lines represented high and low EMT risk score groups, separately. **(D)** The area under curves (AUCs) plot of EMT risk score in TCGA-BLCA cohort. **(E, F)** K-M plot of OS between EMT risk score groups and AUCs plot of the risk score the in Xiangya validation cohort, separately. **(G, H)** K-M plot of OS between EMT risk score groups and AUCs plot of the risk score in the GSE13507 validation cohort, separately. **(I, J)** K-M plot of OS between EMT risk score groups and AUCs plot of the risk score in GSE48075 validation cohort, separately. Red and light blue lines represented high and low EMT risk score groups, separately.

Firstly, 161 DEGs were screened out between the two EMT clusters using the “limma” R package (**Supplementary Table S7**). Univariate Cox analysis helped us further screen 70 genes related to prognosis from these 161 DEGs (**Supplementary Table S8**). Subsequently, we further refined these 70 prognostis related DEGs suitable for modeling by constructing LASSO regression model. Moreover, we selected the minimum lambda value (0.11) as the best node, under the 10-fold cross-validation, and finally selected 7 candidate genes (**Figures 3A, B**), including ANXA10, CNTN1, FAM180A, FN1, IGFL2, KANK4 and TOX3. Finally, we used the “randomForestSRC” R package to build a random forest model. Based on the 7 candidate EMT genes, we develop a risk score in TCGA-BLCA, named EMT risk score. We used the median score as the criterion to divide the cohort into high EMT risk score and low EMT risk score.

The difference in prognosis of the training cohort was shown in **Figure 3C**. The OS of patients with high EMT risk score was significantly lower than that of low EMT risk score (p<0.0001), which suggested that EMT risk score was a risk factor for prognosis of BLCA. In addition, the accuracy of EMT risk score in predicting OS in 1, 3 and 5 years was 0.95, 0.95 and 0.94 separately (**Figure 3D**).

Then we validated this EMT risk score in external cohorts, including Xiangya, GSE13507, GSE48075 cohort. In the Xiangya cohort (**Figures 3E, F** **)**, patients with high EMT risk scores had a worse prognosis (p=0.0046), and the AUCs for predicting 1, 2 and 3-year OS were 0.75, 0.71 and 0.88, separately. In GSE13507 (**Figures 3G, H** **)**, the prognosis of patients with high EMT risk score was still worse than those with low EMT risk score (p=0.0093), and the AUCs of 1, 3 and 5-year OS were 063, 0.66 and 0.67, separately. The same results still appeared in GSE48075 (**Figures 3I, J** **)**. Patients with high EMT risk score showed lower OS (p=0.0057), and their AUCs predicting 1, 3 and 5-year prognosis were 0.62, 0.65 and 0.52, separately. The above results were highly consistent with the training cohort, indicating that the EMT risk score had good external generalization, that is, high external authenticity, and could be further studied.

### Construction of a nomogram by integrating the EMT risk score and clinicopathological features in TCGA-BLCA cohort

**Figure 4:**
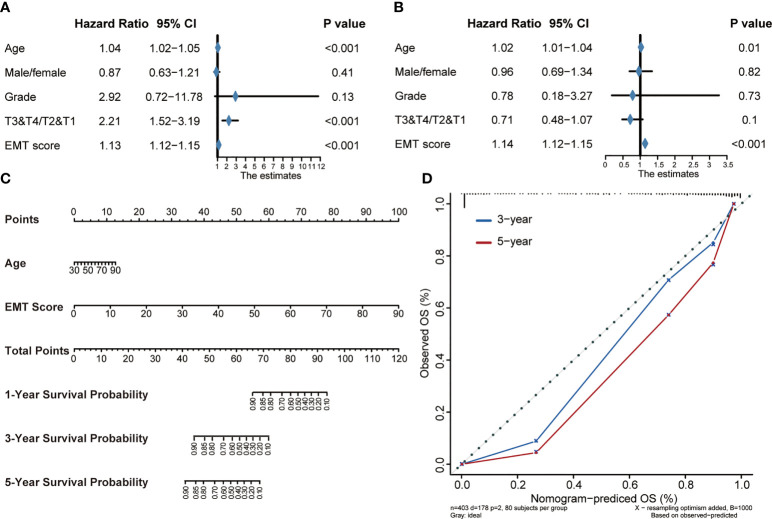
Construction of a Nomogram by integrating the EMT risk score and clinicopathological features in TCGA-BLCA cohort. **(A, B)** Forest plots of univariate and multivariate Cox analysis of TNF-based risk score combined with age, gender, tumor grade and stage of BLCA. **(C)** Nomogram developed by using age and EMT risk score. **(D)** Calibration curves of the nomogram.

In order to further study the clinical application value of EMT risk score, we studied EMT risk score as an independent clinical index together with other clinical information. Univariate Cox analysis was performed on EMT risk score, age, gender, tumor grade and stage of BLCA, as shown in **Figure 4A**, EMT risk score was an important risk factor affecting prognosis (p<0.001). Then, multivariate Cox analysis showed that EMT risk score was still an independent prognostic factor (p<0.001) (**Figure 4B**). These results confirmed that EMT risk score was likely to be a potentially valuable prognostic indicator for BLCA. Therefore, we combined the EMT risk score with other clinical information of independent predictors suggested by multivariate Cox analysis, including age, to construct a nomogram (**Figure 4C**). In the nomogram, EMT risk score had a great contribution to the prediction of survival probability. As shown in the calibration curves (**Figure 4D**), the OS predicted by the nomogram was basically consistent with the actual OS, which highlighted that the nomogram had great reliability and value in clinical application.

### Association between EMT risk score and TIME in the TCGA-BLCA and Xiangya cohort

**Figure 5:**
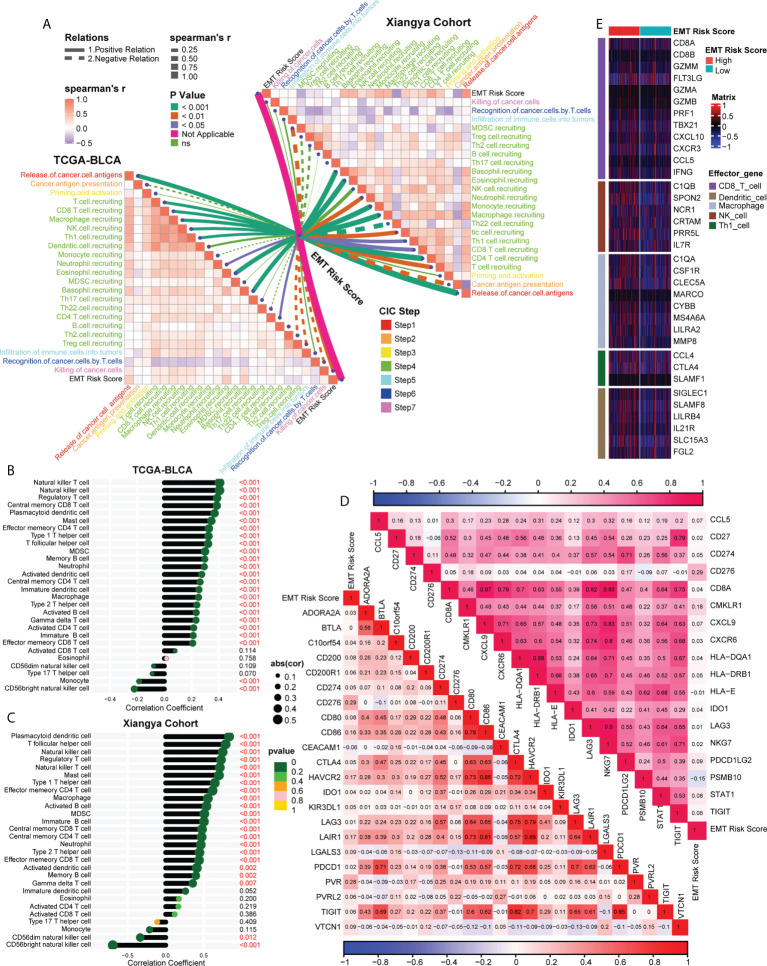
Association between EMT risk score and TIME in the TCGA-BLCA and Xiangya cohort. **(A)** The association between EMT risk score and cancer immunity cycles in the TCGA-BLCA (left) and Xiangya cohort (right). The different types of lines represented the positive or negative relations; The different colors of the lines represented the p values of the relations, and the thickness of the lines represented the strength of the relations. **(B, C)** The association between EMT risk score and immune cells in the TME in the TCGA-BLCA and Xiangya cohort. **(D)** The association between EMT risk score and immune checkpoint inhibitor (ICI) genes (down) and T cell-associated inflammatory signature (TIS) genes (up), separately. **(E)** The different expression patterns of effector genes of immune cells between different EMT risk score groups.

Analyzing the characteristics of TIME can help improve the ability to predict and guide immunotherapy response, and reveal new therapeutic targets (23). Therefore, we were interested in studying whether EMT risk score could distinguish the characteristics of TIME of BLCA and guide the choice of treatment. Firstly, in the Cancer Immunity Cycle, the main anti-tumor immune steps, such as step 1 (release of cancer cell antigens), step 4 (T cell recruiting, CD8 T cell recruiting, Macrophage recruiting, NK cell recruiting, Th1 cell recruiting) and step 7 (Killing of cancer cells), showed a significant positive correlation with EMT risk score (**Figure 5A**, left, **Supplementary Table S9**). In addition, we verified the relationship between TIME and EMT risk score in Xiangya cohort. As expected, the EMT risk score was positively correlated with the activation of the main steps of 7-step Cancer-Immunity Cycle (**Figure 5A**, right, **Supplementary Table S10**). Accordingly, as shown in **Figure 5B** (**Supplementary Table S11**), the infiltration level of immune cells including activated CD4 T cell, activated B cell, type 1 T helper cell, natural killer cell and macrophage in TIME was also apparently positively correlated with EMT risk score. In Xiangya corhort, the corresponding immune cells in TIME were also higher in patients with high EMT risk score (**Figure 5C**, **Supplementary Table S12**). Further, we found that the expression of most ICI genes and TIS genes were positively correlated with EMT risk score (**Figure 5D**, **Supplementary Table S13**). As shown in **Figure 5E**, the expression of genes representing the effects of immune cells, including dendritic cells, macrophages, NK cells and Th1 cells, was higher in high EMT risk score than in low EMT risk score. Therefore, compared with EMT expression pattern, EMT risk score could more effectively quantitatively evaluate the TIME phenotype of a single patient and estimate the treatment response rate of immunotherapy.

### EMT risk score predicted molecular subtypes of MIBC in the TCGA-BLCA and Xiangya cohort

MIBC could be stratified into different molecular subtypes, and the different subtypes also suggested distinct prognosis and treatment response (8). However, the classification criteria of molecular subtypes were various, which hinders its clinical application. Therefore, combined with our previous research (20), seven molecular typing standards, including TCGA subtype (24), MDAnderson Cancer Center (MDA) subtype (25), Lund subtype (26), Cartes d’Identité des Tumeurs-Curie (CIT) subtype (27), University of North Carolina (UNC) subtype (28), Baylor subtype (29) and consensus subtype (21), were integrated for comprehensive correlation analysis.

**Figure 6:**
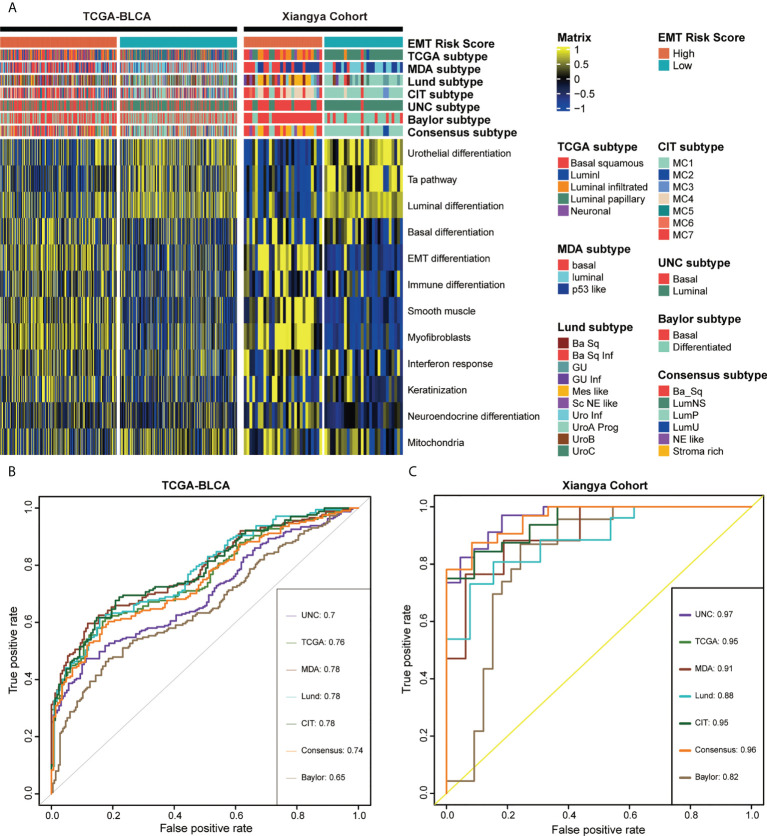
EMT risk score predicted molecular subtypes of MIBC in the TCGA-BLCA and Xiangya cohort. **(A)** The heatmap of different EMT risk score groups, seven molecular subtype classifications and bladder cancer associated signatures in the TCGA-BLCA (left) and Xiangya cohort (right). Activated or inhibited pathways are marked as yellow or blue, separately. **(B, C)** ROC plot of the EMT risk score for predicting seven molecular subtype classifications in BLCA in the TCGA-BLCA and Xiangya cohort.

As shown in **Figure 6A**, left, in the TCGA-BLCA cohort, we found that the high EMT risk score group generally represented the basal subtype and showed some activated biological characteristics such as basal differentiation, EMT differentiation, Immune differentiation, myofibroblasts, interferon response, and so on. In contrast, the low EMT risk score group indicated the luminal subtype which was characterized by the activation of urothelial differentiation, Ta pathway and luminal differentiation. Moreover, EMT risk score had high accuracy in predicting BLCA molecular subtypes, and most AUCs were above 0.75 (**Figure 6B**). Consistent with the expected results, in our own Xiangya cohort (**Figure 6A**, right), patients with high EMT risk scores suggested basal subtype, while those with low EMT risk scores represented luminal subtype, and both groups showed their respective biological characteristics. Moreover, most of the AUCs that EMT risk scores predicted molecular subtypes exceeded 0.90 (**Figure 6C**).

Compared with the luminal subtype, the TME of the basal subtype BLCA had more immune cell infiltration and better curative effect on neoadjuvant chemotherapy (NAC) and immunotherapy (21, 30). The above results showed that patients with high EMT risk scores represented basal subtypes, and were more likely to express inflammatory TIME and had a higher response rate to immune checkpoint blockade (ICB).

## Discussion

About 1/4 of BLCA patients were found that the tumor tissue had infiltrated into the muscular layer at the time of diagnosis, and those diagnosed with MIBC tended to have faster cancer progression, poorer prognosis (3) and heavier financial burden. Current studies have proved that EMT was not a binary process, but a plasticity process regulated by gene network that gradually affects tumor microenvironment and metastasis (13). Rhim, A. D. et al. found that the promotion of EMT on tumor metastasis occurred in the early stage of cancer, even in precancerous lesions or before tumor formation, and the occurrence and development of EMT was promoted by inflammation (31). Therefore, we were interested in studying the value of EMT in prognosis prediction and immune microenvironment differentiation of BLCA.

First of all, we found that most of the prognosis related EMT genes suggest a poor prognosis of BLCA, and there is a significant correlation between them. We integrated the expression characteristics of 200 EMT related genes, and developed two EMT clusters with consensus clustering. We found that there were significant differences in the prognostic outcome, clinical characteristics and immune cell infiltration level of different clusters. The prognosis and biological characteristics of cancer cannot be simply determined by the cancer cells, but the impact of TME on tumor occurrence, progression and therapeutic intervention must be considered (23). Duan, Q et al. suggested that T cell infiltration and activation or deletion and rejection can divide TME into hot and cold (11), while the two EMT expression patterns in our study can significantly distinguish hot or cold TME.

Gene expression patterns can not meet the needs of precision medicine for cancer patients, therefore, some previous studies, such as Cao R et al. (32) and Wang L (33), had developed quantitative scores to preliminarily explore the prognostic value of EMT-related genes in BLCA, but they did not comprehensively combine EMT with prognosis, immunity and molecular subtype of BLCA. In this study, we screened and refined the EMT pathway related genes and developed an EMT-related risk score, which was the first comprehensive quantitative assessment of the prognostic value of EMT in predicting the prognosis, molecular subtype and TIME of BLCA.

Our EMT risk score and EMT related nomogram could predict the prognosis and outcome of a single patient, with robust accuracy and generalization. Dudas, J et al. (34) systematically summarized the drug resistance mechanism of EMT, including signal pathways (TGF-β1, IL-6, Akt, and Erk1/2), RNA molecules (RNA molecules without protein translation, micro-RNAs, and lnc-RNAs), induced cells (fibroblasts and myofibroblasts) and some EMT inhibition mechanisms. TGF-β 1 was considered to play a two-way role in tumorigenesis, that was, inhibit tumorigenesis in early cancer, but promote cancer progression (35, 36). Therefore, TGF-β 1 was considered to be an important mediator of EMT. EMT transcription factors (EMT-TFs) could promote the movement and proliferation of tumor cells, which was a key event in EMT (37). And EMT TFs in cancer tissue, such as SNAIL, ZEB, and TWIST, significantly indicated a poor prognosis outcome (37, 38). Cancer associated fibroblasts (CAFs) could directly promote tumor cells to secrete cytokines such as interleukin 6 (IL-6) and TGF-β 1 (39, 40), induce the occurrence of EMT, and promote the proliferation and invasion of tumor cells (41). In addition, TGF could also promote smooth muscle actin to promote the transformation of fibroblasts into myofibroblasts and enrich the composition of CAFs (42). The targeted therapeutic agent of WNT signaling pathway (participating in EMT) had entered the stage of clinical trial, and porcupine (PORCN) inhibitor LGK-974 can inhibit the growth of pancreatic cancer cells (43, 44).

The classification of tumor-infiltrating immune cells (TICs) and the functional interaction between TICs and cancer cells can regulate the development of cancer (45). There was ample evidence that TICs in TME, including CD8 positive T cells and NK cells, were not only related to the prognosis of BLCA, but also a promising biomarker prediction tool for treatment response (12). Moreover, Suarez-Carmona M, et al. concluded that EMT tumor cells can induce infiltrating inflammatory cells and promote cancer metastasis, such as inducing “M2” polarization of macrophages infiltrating TME (46). Cancer immunotherapy has become a hot topic, such as targeted metabolism to improve the tumor microenvironment (46), so the deep understanding of TIME needs to be deepened all the time. More and more researches were devoted to developing markers or models that can identify the phenotype of immune microenvironment (47, 48). Our EMT risk score was the first model developed based on the expression characteristics of EMT-related genes. It could systematically obtain consistent and robust results in predicting prognosis, immune microenvironment and molecular typing, and the results have been well verified in external cohorts, especially in our hospital cohort (Xiangya cohort).

The topic that MIBC could be divided into different molecular subtypes according to the gene expression characteristics has been widely studied. Molecular typing might predict the prognosis (49) and the response to treatments such as neoadjuvant chemotherapy (30) and immune checkpoint inhibitors (50) of bladder cancer. And studies have shown that the molecular subtype may also be heterogeneous (51). Thus, molecular typing of bladder cancer was potentially important in predicting prognosis and guiding the choice of treatment modalities. More and more studies focus on the classification of molecular subtypes of cancer from more levels, such as premalignant lesions (PMLs) (52), to promote precision medicine. There were many ways of molecular typing, such as TCGA, MDA, UNC, and consensus subtypes, and BLCA was mainly divided into basal subtype, luminal subtype and others. Previous studies had shown that, compared with luminal subtype, basal subtype had worse prognosis (25) but higher level of immune cell infiltration (53) and higher response rate to immunotherapy and NAC (30). In order to overcome the application obstacles caused by different molecular typing standards, we systematically synthesized seven molecular typing methods based on the previous research of our team (20). In this study, EMT risk score can effectively divide BLCA patients into basial and luminal subtypes, and the prognosis and immune cell infiltration results of the two subtypes were consistent with previous studies. In conclusion, our results verified tumor immunity again from the perspective of molecular subtype.

Finally, there are some limitations of this study, some of which need to be further improved and deepened in the future. For example, the cohorts used in our study were retrospective cohorts, but the results could be used as expectations to plan further prospective studies. In addition, the independent value of EMT candidate genes for MIBC in this study had not been further explored, and our team is conducting follow-up research. Moreover, tissue section observation, cell experiment and animal experiment are also taken into account in the future research design to further confirm the mechanism of EMT pathway on MIBC. And further exploring the value of EMT-based risk score in immunotherapy and clinical drugs screening will also be considered.

## Conclusion

EMT related genes play an important role in tumor progression and immunity in BLCA. Our EMT risk score could accurately predict prognosis and immunophenotype of a single patient, which could guide more effective precision medical strategies.

## Data availability statement

Publicly available datasets were analyzed in this study. This data can be found here: https://www.ncbi.nlm.nih.gov/geo/GSE188715.

## Author contributions

ZX, ZC and DD performed analyses and drafted the manuscript. ZX and ZC searched and downloaded the original datasets from TCGA and GEO. ZX and ST contributed to statistical analyses. ZX, ZC, DD, ST and ZX edited the pictures. ZX and ST conceived and supervised the study. All authors contributed to the article and approved the submitted version.

## Funding

This work was supported by the National Natural Science Foundation of China (81873626, 81902592, 82070785), Hunan Natural Science Foundation (2020JJ5884) and Hunan Province Young Talents Program (2021RC3027).

## Acknowledgments

We sincerely thank all participants in the study.

## Conflict of interest

The authors declare that the research was conducted in the absence of any commercial or financial relationships that could be construed as a potential conflict of interest.

## Publisher’s note

All claims expressed in this article are solely those of the authors and do not necessarily represent those of their affiliated organizations, or those of the publisher, the editors and the reviewers. Any product that may be evaluated in this article, or claim that may be made by its manufacturer, is not guaranteed or endorsed by the publisher.

## Supplementary material

Supplementary Table 1 The clinical information of the TCGA-BLCA, Xiangya, GSE13507, and GSE48075 cohorts.

Supplementary Table 2 200 EMT-related genes included in this study.

Supplementary Table 3 The gene set for calculating immune cells in the TIME using the ssGSEA algorithm.

Supplementary Table 4 22 ICI genes, 18 TIS genes and effector genes of immune cells, separately.

Supplementary Table 5 Gene signatures that are closely associated with molecular subtypes of BLCA.

Supplementary Table 6 GO, KEGG and HALLMARK pathways results of the difference between EMT cluster 1 and cluster 2; EMT Cluster1 vs EMT Cluster2.

Supplementary Table 7 161 DEGs between EMT cluster 1 and cluster 2 using the “limma” R package.

Supplementary Table 8 The univariate prognostic values of the 70 EMT-related genes.

Supplementary Table 9 The association between EMT risk score and cancer immunity cycles in TCGA-BLCA cohort.

Supplementary Table 10 The association between EMT risk score and cancer immunity cycles in Xiangya cohort.

Supplementary Table 11 The association between EMT risk score and immune cells in the TIME in TCGA-BLCA cohort.

Supplementary Table 12 The association between EMT risk score and immune cells in the TIME in Xiangya cohort.

Supplementary Table 13 The association between EMT risk score, ICI genes and TIS genes in the TCGA-BLCA cohort.

## Abbreviations

EMT, Epithelial mesenchymal transition; TME, tumor microenvironment; BLCA, bladder cancer; LASSO, least absolute shrinkage and selection operator; OS, overall survival; MIBC, muscle-invasive bladder cancer; NMIBC, non-muscle-invasive bladder cancer; TIME, tumor immune microenvironment; GRN, gene regulatory network; TCGA, The Cancer Genome Atlas; GDC, Genomic Data Commons; FPKM, fragments per kilobase per million mapped fragments; TPM, transcripts per kilobase million; RNA-seq, RNA-sequencing; GEO, Gene Expression Omnibus; DEG, differential expressed gene; FC, fold change; GO, Gene Ontology; KEGG, Kyoto Encyclopedia of Genes and Genomes; TIP, tracking tumor immunophenotype; ssGSEA, single-sample gene set enrichment analysis; ICI, immune checkpoint inhibitor; TIS, T cell-associated inflammatory signature; DC, dendritic cell; NK cell, natural killer cell; Th1 cell, Type 1 T helper cell; RSF, random survival forest; ROC, receiver operating characteristic; AUC, area under the curve; K-M, Kaplan-Meier; HR, hazard ratio; LVI, lymphatic vessel invasion; GSVA, gene set variation analysis; MDA, MDAnderson Cancer Center; CIT, Cartes d’Identité des Tumeurs-Curie; UNC, University of North Carolina; ICB, immune checkpoint blockade; NAC, neoadjuvant chemotherapy; PD-1 and PD-L1, programmed cell death-1 and its ligand.
